# Ethanol-Dependent Synthesis of Salsolinol in the Posterior Ventral Tegmental Area as Key Mechanism of Ethanol’s Action on Mesolimbic Dopamine

**DOI:** 10.3389/fnins.2021.675061

**Published:** 2021-06-28

**Authors:** Valentina Bassareo, Roberto Frau, Riccardo Maccioni, Pierluigi Caboni, Cristina Manis, Alessandra T. Peana, Rossana Migheli, Simona Porru, Elio Acquas

**Affiliations:** ^1^Center of Excellence for the Study of Neurobiology of Addiction, University of Cagliari, Cagliari, Italy; ^2^Department of Biomedical Sciences, University of Cagliari, Cagliari, Italy; ^3^Department of Life and Environmental Sciences, University of Cagliari, Cagliari, Italy; ^4^Department of Chemistry and Pharmacy, University of Sassari, Sassari, Italy; ^5^Department of Experimental Medical and Surgical Sciences, University of Sassari, Sassari, Italy

**Keywords:** acetaldehyde, brain microdialysis, dopamine, ethanol, μ opioid receptors, nucleus accumbens shell, posterior ventral tegmental area, salsolinol

## Abstract

Abnormal consumption of ethanol, the ingredient responsible for alcoholic drinks’ addictive liability, causes millions of deaths yearly. Ethanol’s addictive potential is triggered through activation, by a still unknown mechanism, of the mesolimbic dopamine (DA) system, part of a key motivation circuit, DA neurons in the posterior ventral tegmental area (pVTA) projecting to the ipsilateral nucleus accumbens shell (AcbSh). The present *in vivo* brain microdialysis study, in dually-implanted rats with one probe in the pVTA and another in the ipsilateral or contralateral AcbSh, demonstrates this mechanism. As a consequence of the oral administration of a pharmacologically relevant dose of ethanol, we simultaneously detect a) in the pVTA, a substance, 1-methyl-6,7-dihydroxy-1,2,3,4-tetrahydroisoquinoline (salsolinol), untraceable under control conditions, product of condensation between DA and ethanol’s first by-product, acetaldehyde; and b) in the AcbSh, a significant increase of DA release. Moreover, such newly generated salsolinol in the pVTA is responsible for increasing AcbSh DA release *via* μ opioid receptor (μOR) stimulation. In fact, inhibition of salsolinol’s generation in the pVTA or blockade of pVTA μORs prevents ethanol-increased ipsilateral, but not contralateral, AcbSh DA release. This evidence discloses the long-sought key mechanism of ethanol’s addictive potential and suggests the grounds for developing preventive and therapeutic strategies against abnormal consumption.

## Introduction

Ethanol, key ingredient of alcoholic drinks, is one of the most used and abused psychoactive substances worldwide and underlies the potential of alcoholic drinks to trigger their abnormal/heavy consumption (Abrahao et al., 2017). In addition, ethanol is a risk factor for other serious illnesses including cancer and liver and cardiovascular diseases (Rehm et al., 2017; Axley et al., 2019).

The molecular mechanism by which ethanol may trigger its excessive ingestion has been the subject of intense research (Koob, 2004; Belmer et al., 2016; Koob and Volkow, 2016). Two seminal observations led to the modern studies on the neurobiology of ethanol. Monoamines are involved in ethanol’s reinforcing and euphoriant effects (Ahlenius et al., 1973), and ethanol activates the ventral tegmental area (VTA) mesolimbic DA neurons (Gessa et al., 1985). Ensuing research demonstrated that ethanol’s reinforcing properties are mediated by actions on the mesolimbic DA system (Di Chiara et al., 2004; Howard et al., 2008; Bassareo et al., 2017, 2019; Volkow et al., 2017; Wise and Robble, 2020), but the underlying mechanism and molecular target have remained not fully understood. However, as extensively discussed in a recent review (Sanchez-Catalan et al., 2014), a pivotal role is recognized to DA neurons in the posterior, but not anterior, ventral tegmental area (pVTA) (Rodd et al., 2004; Hauser et al., 2011; Ding et al., 2012) and their projections (Ungerstedt, 1971) to the nucleus accumbens shell (AcbSh) (Di Chiara et al., 2004). These neurons are a key component of a brain motivation circuit activated by addictive drugs (Di Chiara et al., 2004; Volkow et al., 2017; Wise and Robble, 2020). Ethanol is no exception to this general framework, and the role of ethanol metabolism in the pVTA, in this regard, has been emphasized. Accordingly, already in the early 1950s, acetaldehyde, the main ethanol’s metabolite, was suggested as able to mediate ethanol’s reinforcing effects (Chevens, 1953). This hypothesis generated a significant body of research aimed at characterizing the role of ethanol metabolism in its central effects (Karahanian et al., 2011; Correa et al., 2012), in particular with reference to its ability to activate the mesolimbic DA system (Correa et al., 2012; Söderpalm and Ericson, 2013; Israel et al., 2015). Likewise, this evidence suffers a lack of general consensus (Söderpalm and Ericson, 2013) and of a specific molecular mechanism (Israel et al., 2015; Peana et al., 2017b, 2019). This notwithstanding, the ability of ethanol to stimulate DA neurons in the pVTA (Gessa et al., 1985), activate DA transmission in the AcbSh (Howard et al., 2008; Bassareo et al., 2017), and elicit DA-mediated locomotor activity (Carlsson et al., 1972; Sánchez-Catalán et al., 2009) has been mechanistically related to ethanol’s main metabolite, acetaldehyde. Such role of acetaldehyde has been demonstrated by two independent lines of evidence: first, acetaldehyde administration reproduces, at lower doses than ethanol, DA-dependent central effects (Spina et al., 2010; Correa et al., 2012); second, inhibition of acetaldehyde production, by blockade of peripheral and central ethanol metabolizing enzymes (Spivak et al., 1987; Pastor et al., 2002; Peana et al., 2017a) or by acetaldehyde sequestration (Martí-Prats et al., 2013), prevents both neurochemical and behavioral DA-mediated effects of ethanol. The mechanism(s) by which acetaldehyde exerts such effects is unknown (Peana et al., 2017b). Notably, acetaldehyde has a very short half-life and is highly reactive with molecules such as DA. Indeed, although an enzymatic mechanism has also been claimed (Chen et al., 2018), acetaldehyde and DA can spontaneously generate a condensation product, 1-methyl-6,7-dihydroxy-1,2,3,4-tetrahydroisoquinoline or salsolinol, a molecule undetectable, under control conditions, in the VTA. This led to renewed interest in ethanol’s secondary metabolites (Davis and Walsh, 1970; Davis et al., 1970; Rodd et al., 2008; Hipólito et al., 2012; Deehan et al., 2013; Israel et al., 2015; Quintanilla et al., 2016). Intriguingly, salsolinol, administered either systemically or locally in the pVTA, shares with ethanol and acetaldehyde a spectrum of neurochemical and behavioral DA-mediated effects (Correa et al., 2012; Hipólito et al., 2012; Deehan et al., 2013). Moreover, salsolinol is self-administered by rats in the pVTA (Rodd et al., 2008), and its pVTA administration promotes voluntary binge-like ethanol intake (Quintanilla et al., 2016).

Notwithstanding, to date, the hypothesis of ethanol as the prodrug of salsolinol for the activation of the mesolimbic DA system has not been directly addressed *in vivo* (Polache and Granero, 2013; Peana et al., 2016). The present study aimed at challenging this hypothesis. To this end, we applied *in vivo* brain microdialysis to freely behaving rats implanted two microdialysis probes, one in the pVTA and another in the AcbSh of either the same side (ipsilateral) or of the opposite side of the brain (contralateral). We hypothesized that the systemic administration of a dose of ethanol known to result in pharmacologically and clinically relevant blood ethanol concentrations (BEC) (Gill et al., 1986; Majchrowicz, 1975; Nurmi et al., 1994) could result in pharmacologically meaningful salsolinol concentrations in the pVTA and in salsolinol-dependent increases of DA release in the AcbSh. In fact, since the prevailing VTA–AcbSh projections are ipsilateral (Ikemoto, 2007; Breton et al., 2019) with only ∼5% of midbrain neurons projecting contralaterally (Jaeger et al., 1983; Geisler and Zahm, 2005), we planned to explore the impact of preventing the conversion of ethanol into salsolinol only in the pVTA of one side (either left or right). We reasoned that applying by reverse dialysis in the pVTA of one side either an acetaldehyde-sequestering agent (thus reducing acetaldehyde’s availability) (Martí-Prats et al., 2013) or a catalase inhibitor (thus preventing catalase-mediated oxidation of ethanol into acetaldehyde (Pastor et al., 2002) or a DA D_2_/D_3_ receptor agonist (thus reducing synaptic availability of DA) (Kohl et al., 1998) would prevent salsolinol formation and hence ethanol’s effects on DA release in the ipsilateral, but not in the contralateral, AcbSh. Finally, based on the evidence that both the neurochemical and the behavioral pVTA-dependent effects of ethanol and salsolinol (Hipólito et al., 2011; Xie et al., 2012) are mediated by μ opioid receptors (μORs), we tested the hypothesis that ethanol-derived salsolinol in the pVTA increases DA release in the AcbSh *via* a μOR-mediated mechanism.

## Materials and Methods

### Animals

Male Sprague–Dawley rats (275–325 g) (Envigo, Desio, Italy) were used for the experiments. Animals had access to water and standard food (Stefano Morini, S. Polo D’Enza, RE, Italy) *ad libitum* and were handled throughout the experimental procedures in accordance with the guidelines for the care and use of experimental animals of the European Community Council (2010/63/UE L 276 20/10/2010) and with Italian law (DL 04.03.2014, *N*° 26; Authorization *n*° 1177/2016). Every effort was made to minimize suffering and reduce the number of animals used.

### Surgery

Beginning 3 days before surgery, rats were handled once daily for 5 min to habituate them to the experimental procedures [intraperitoneal (i.p.) and intragastric (i.g.) administrations]. Under deep anesthesia by equitesin (0.97 g pentobarbital, 4.25 g chloral hydrate, 2.1 g MgSO_4_, 42.8 ml propylene glycol, 11.5 ml 90% ethanol/100 ml; 5 ml/kg i.p.) (Bassareo et al., 2019), vertical microdialysis probes were stereotaxically implanted in the pVTA and in the AcbSh using the following coordinates according to Paxinos and Watson (1998): AP: –5.8 mm and ML: ± 0.5 mm from bregma and DV: –8.0 mm from dura, for the pVTA; AP: 1.8 mm and ML: ± 1 mm from bregma and DV: –7.6 mm from dura, for the AcbSh. After surgery, rats were housed in individual hemispheric cages under the same standard conditions, left undisturbed for at least 24 h, and fed with 20 g of standard chow (their weight being maintained at ∼95% of their *ad libitum* weight). Water was available *ad libitum* throughout, except during the microdialysis experiments.

### Probe Preparation

Vertical dialysis probes (dialyzing portion: 1.5 mm; outer diameter: 300 μm) were prepared with AN69 fibers (Bassareo et al., 2019) (Hospal-Dasco, Bologna, Italy) for both the pVTA and the AcbSh. In the case of pVTA, however, we acknowledge that such length of the dialyzing portion may have extended dorsally outside the anatomical border of the targeted area.

### Drugs and Treatments

Ethanol, diluted at the concentration of 20% (v/v) in tap water (vehicle), was administered intragastrically (i.g.) at the dose of 1 g/kg/10 ml. (±)-Salsolinol (Santa Cruz Biotechnology Inc., Dallas, TX, United States) was dissolved in normal Ringer (see below) to 10 nM and used in place of normal Ringer to perfuse the microdialysis probe in the pVTA. D-penicillamine (DP), an acetaldehyde-sequestering agent; 3-amino-1,2,4-triazole (3AT), a non-competitive catalase inhibitor; quinpirole, a dopamine D_2_/D_3_ receptor agonist; and naltrexone, a μ opioid receptor antagonist, were dissolved in normal Ringer at 75 μM (DP), 1 nM (3AT and naltrexone), and 2.5 μM (quinpirole) and were used in place of normal Ringer toperfuse the microdialysis probe in the pVTA of one side (either left or right) starting 30 min before the administration of vehicle or ethanol. pVTA perfusion with salsolinol, DP, 3AT, quinpirole, or naltrexone was maintained until the end of the microdialysis experiment. Ethanol, DP, 3AT, quinpirole, and naltrexone were purchased from Sigma-Aldrich, Milan, Italy. The dose of ethanol (Howard et al., 2008; Bassareo et al., 2019) and the concentrations of salsolinol (Hipólito et al., 2009, 2011), DP (Orrico et al., 2017), 3AT (Rodd et al., 2005; Melis et al., 2015), quinpirole (Rodd et al., 2004; Hauser et al., 2011), and naltrexone (Latagliata et al., 2014) for pVTA reverse dialysis were selected based on previous literature.

### Microdialysis Experiments

**FIGURE 1:**
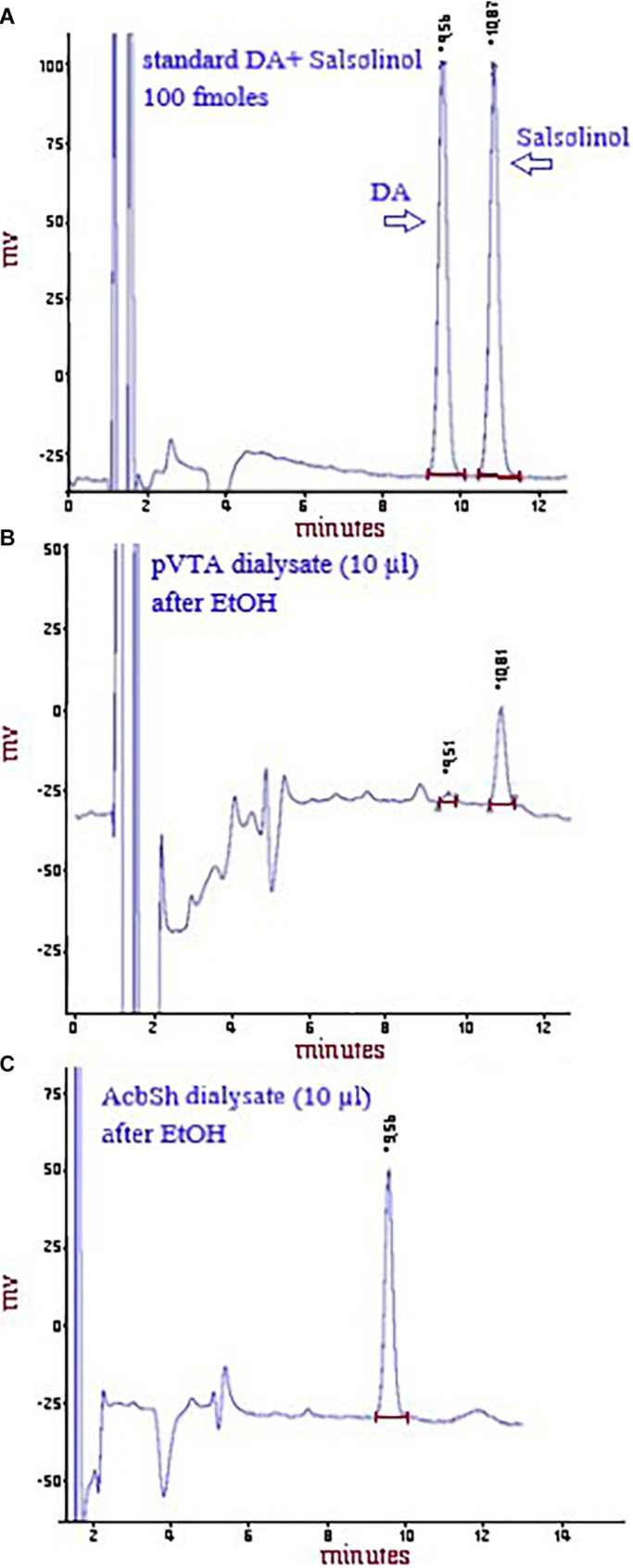
Representative chromatograms showing the peaks of DA and salsolinol after the HPLC injection of a standard solution of 100 fmol of both DA and salsolinol/10 μl **(A)** or of DA and salsolinol after the HPLC injection of a pVTA dialysate (10 μl) from a rat administered ethanol **(B)** or of DA, but not salsolinol (see sections “Materials and Methods” and “Discussion” and Zimatkin and Lindros, 1996) after the HPLC injection of an AcbSh dialysate (10 μl) from a rat administered ethanol **(C)**.

On the experiment day, in awake and freely moving animals, pVTA and AcbSh probes were connected to an infusion pump and perfused with normal Ringer solution (in mM: 147 NaCl, 4 KCl, 2.2 CaCl_2_) at a constant flow rate of 1 μl/min, and when for technical reasons this could not be guaranteed, the experiment was dropped. Dialysate samples (10 μl) were injected without purification into a high-performance liquid chromatograph (HPLC) equipped with a reverse-phase column (LC-18 DB, 15 cm, 5 μm particle size, Supelco-Waters, Milford, MA, United States) and a coulometric detector (ESA-Coulochem II, Bedford, MA, United States) to simultaneously quantify salsolinol and DA (Figure 1). The first electrode of the detector was set at +125 mV (oxidation) and the second at –175 mV (reduction). The composition of the mobile phase was as follows (in mM: 50 NaH_2_PO_4_, 0.1 Na_2_-EDTA, 0.5 n-octyl sodium sulfate, and 15% (v/v) methanol, pH 5.5 obtained adding Na_2_HPO_4_). Under these conditions, the sensitivity of the assay for salsolinol and DA in the pVTA and DA in the AcbSh was 5 femtomoles (fmol)/sample). DA concentration in pVTA dialysates was often erratic due to its value often below the detection limit of our analytical systems, and for this reason, it was not assayed. Salsolinol was undetectable in AcbSh dialysates, both under basal conditions and after administration of ethanol, in agreement with the lack of catalase’s expression in the nucleus accumbens (Zimatkin and Lindros, 1996). Every subject was utilized for only one microdialysis experiment. Salsolinol and DA data were calculated and plotted as the average ± SEM of the same time points of subjects from each experimental group, before and after changes of perfusion fluid and/or systemic administrations.

### Histology

**FIGURE 2:**
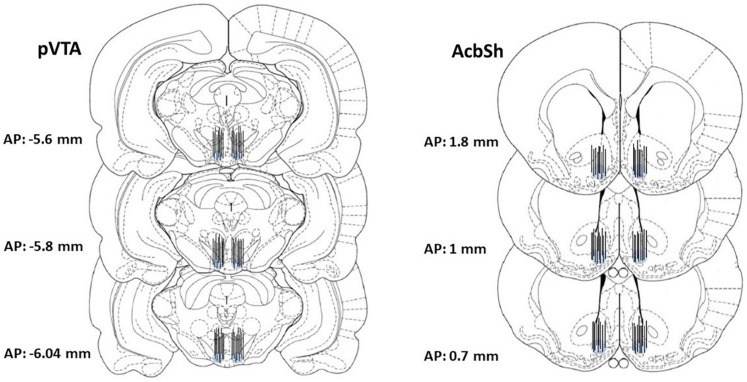
Representative images of the localization, within the pVTA and the AcbSh, of the dialyzing portion of dialysis probes drawn, after histological examination, in the brain atlas plates showing the pVTA and the AcbSh at different AP distances from bregma according to the rat brain atlas of Paxinos and Watson (1998).

At the end of the microdialysis experiment, rats were anesthetized as previously reported (Bassareo et al., 2019), probes were removed, and brains were kept in a 4% (w/v) formaldehyde solution for at least 1 week and successively cut with a vibratome in serial coronal slices oriented according to the rat brain atlas of Paxinos and Watson (1998). The location of the probes was reconstructed and referred to the rat brain atlas plates (Paxinos and Watson, 1998; Figure 2). Data from three rats were excluded for probe misplacement.

### Blood Ethanol Concentration

One hour after vehicle or ethanol administration to rats of separate experimental groups, rats were deeply anesthetized and sacrificed for trunk blood sample (2 ml) collection into heparinized (2,000 UI/2 ml) centrifuge tubes. pVTA-salsolinol and AcbSh-DA data from these animals are not shown (*N* = 8). Quantitative analysis of ethanol was performed by GC-FID according to a previously published method (Penton, 1985) and data were expressed as average ± SEM g/L.

### Statistics

The statistical analysis was carried out by Statistica 8.0 (StatsSoft Inc., Tulsa, OK, United States) for Windows. Basal dialysate salsolinol and DA were calculated as the average ± SEM of the last three consecutive samples differing by no more than 10%, collected during the time period preceding each treatment. Changes in dialysate salsolinol and DA were expressed as fmol/10 μl of dialysate and were analyzed by two- or three-way analysis of variance (ANOVA) with repeated measures over time. Salsolinol data, in addition, in order to take into account the fact that data were under a non-normal distribution, were also analyzed under the Friedman’s ANOVA and Kendall’s coefficient of concordance non-parametric test (general linear model). BEC data were analyzed by one-way ANOVA. Results from treatments showing significant overall changes were subjected to Tukey’s *post hoc* tests with statistical significance set at *p* < 0.05.

## Results

### Effects of Ethanol on pVTA Salsolinol, AcbSh DA, and BEC

**FIGURE 3:**
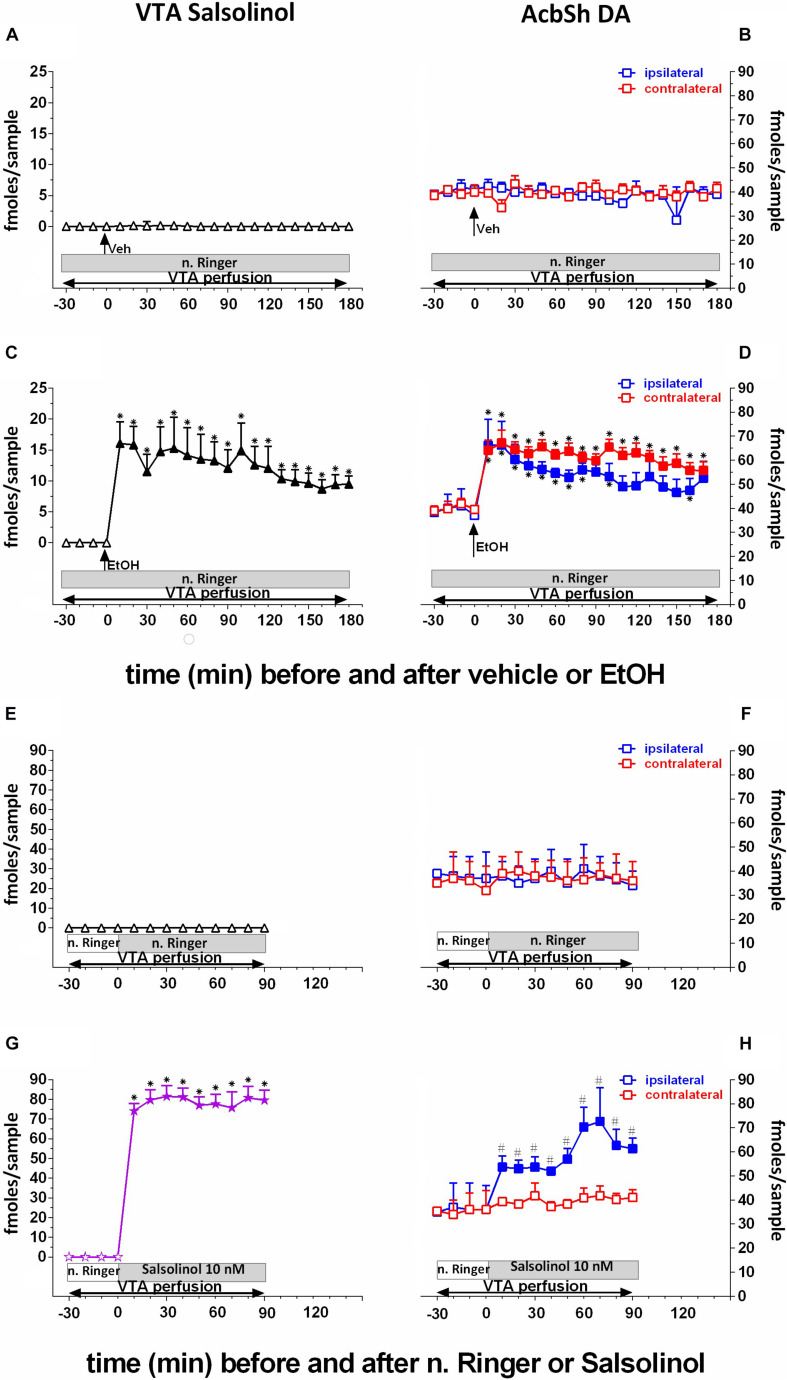
Effects of intragastric administration of vehicle (tap water, 10 ml/kg) [**(A)** *N* = 6, **(B)** *N* = 8] or ethanol (EtOH) (1 g/kg, 20% v/v) [**(C)** *N* = 11, **(D)** *N* = 11] or of reverse dialysis application in the pVTA of normal Ringer (*n*. Ringer) [**(E)** *N* = 6, **(F)** *N* = 8] or salsolinol (10 nM) [**(G)** *N* = 9, **(H)** *N* = 9], on pVTA salsolinol **(A,C,E,G)** and on ipsilateral and contralateral AcbSh DA **(B,D,F,H)** dialysates. The purple color was used here to highlight the fact that indeed these concentrations of salsolinol were from the reverse dialysis application of salsolinol (10 nM)-enriched n. Ringer. Horizontal bars depict the contents of the pVTA perfusion fluid along the experiments. Vertical arrows indicate the last pVTA or AcbSh sample before vehicle or EtOH administration. Filled symbols indicate samples representing *p* < 0.001 vs. basal; ^∗^*p* < 0.01 vs. vehicle administration; ^#^*p* < 0.01 vs. contralateral area.

Figure 3 shows that the intragastric administration of vehicle (10 ml/kg) does not affect salsolinol concentration in pVTA dialysates (Figure 3A) and basal DA release in the AcbSh (Figure 3B). In contrast, the intragastric administration of ethanol (1 g/kg) determines the appearance, in the pVTA, of salsolinol (Figure 3C), a molecule therein undetectable under control conditions, and affects AcbSh DA release (Figure 3D).

Two-way ANOVA of salsolinol concentrations revealed a significant effect of treatment (EtOH vs. vehicle) [*F*_(1_, _13)_ = 95.98; *p* < 0.001] and time [*F*_(18_, _234)_ = 4.85; *p* < 0.001] and a significant treatment × time interaction [*F*_(18_, _234)_ = 4.72; *p* < 0.001]. Moreover, Friedman’s ANOVA and Kendall’s coefficient of concordance analysis resulted in χ^2^_(__*N* = 17_, _*df* = 18)_ = 96.91, *p* < 0.00001; coeff. of concordance = 0.32; average rank *r* = 0.27.

Three-way ANOVA of DA data disclosed a significant effect of treatment (EtOH vs. vehicle) [*F*_(1_, _16_ = 19.9; *p* < 0.001] and time [*F*_(18_, _288)_ = 3.26; *p* < 0.001] and a significant treatment × time interaction [*F*_(18_, _288)_ = 2.76; *p* < 0.001]. Tukey’s *post hoc* analysis revealed that ethanol significantly increases salsolinol and both ipsilateral and contralateral DA with respect to basal values (*p* < 0.05). Moreover, ethanol administration also results in a BEC of 0.61 ± 0.08 g/L. One-way ANOVA of BEC revealed a significant difference between average BEC from vehicle- and ethanol-treated rats [*F*_(1_, _6)_ = 748.31; *p* < 0.001].

### Effects of pVTA Perfusion With Salsolinol on pVTA Salsolinol and Ipsilateral and Contralateral AcbSh DA

To verify the prevailing ipsilateral pVTA–AcbSh projections, we unilaterally perfused with salsolinol, by reverse dialysis, the pVTA (Figures 3G,H). This affects both salsolinol in pVTA (Figure 3G) and DA concentrations in ipsilateral AcbSh (Figure 3H) dialysates.

Two-way ANOVA of salsolinol concentrations showed a significant effect of pVTA perfusion (normal Ringer vs. salsolinol) [*F*_(1_, _11)_ = 121.63; *p* < 0.001] and time [*F*_(9_, _99)_ = 28.46; *p* < 0.001] and a significant treatment × time interaction [*F*_(9_, _99)_ = 28.37; *p* < 0.001].

Three-way ANOVA of DA data showed a significant effect of pVTA perfusion (normal Ringer vs. salsolinol) [*F*_(1_, _12)_ = 12.47; *p* < 0.005], probe placement (ipsilateral vs. contralateral) [*F*_(1_, _12)_ = 21.88; *p* < 0.0005], and time [*F*_(9_, _108)_ = 4.28; *p* < 0.001] and significant treatment × probe placement [*F*_(1_, _12)_ = 15.63; *p* < 0.002], treatment × time [*F*_(9_, _108)_ = 2.65; *p* < 0.008]], and probe placement × time [*F*_(9_, _108)_ = 3.59; *p* < 0.001] interactions.

Tukey’s *post hoc* analysis revealed that perfusion with salsolinol in normal Ringer caused the appearance of a significant concentration of salsolinol in the pVTA (Figure 3G) and a significant increase of ipsilateral AcbSh DA release with respect to basal values (*p* < 0.05) (Figure 3H). In contrast, unilateral application of salsolinol in the pVTA does not affect DA release in the contralateral AcbSh (Figure 3H), confirming the ipsilateral specificity of the pVTA–AcbSh projection.

### Effects of Prevention of pVTA Salsolinol Formation on Ipsilateral and Contralateral AcbSh DA

To assess whether the unilateral prevention of salsolinol production in the pVTA could affect ethanol-mediated stimulation of DA release in the ipsilateral, but not contralateral, AcbSh, we exploited sequestering newly formed acetaldehyde in the pVTA or blocking catalase-mediated production of acetaldehyde. To this end, the acetaldehyde-sequestering agent, DP (75 μM), or the non-competitive catalase-H_2_O_2_ inhibitor, 3AT (1 nM), was unilaterally applied through the pVTA probe.

**FIGURE 4:**
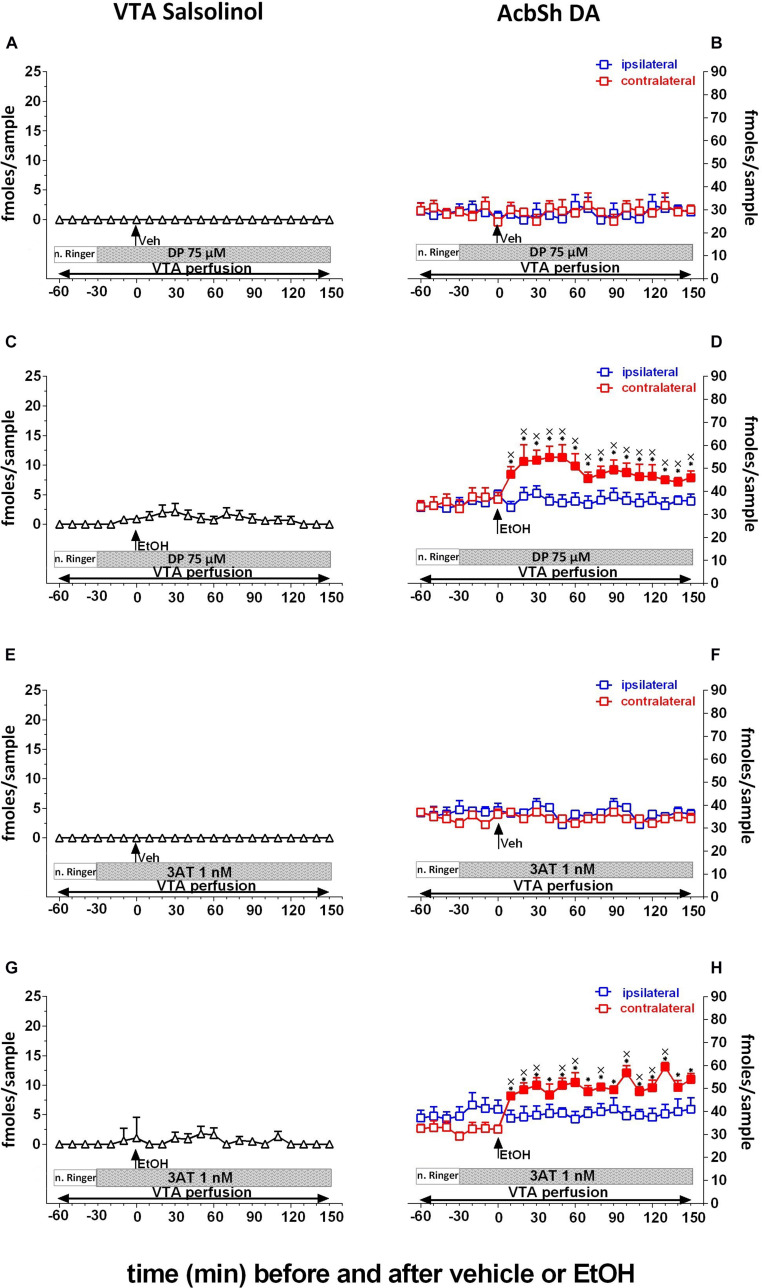
Effects of intragastric administration of vehicle (tap water, 10 ml/kg) [**(A)** *N* = 6, **(B)** *N* = 8, **(E)** *N* = 6, **(F)** *N* = 8] or ethanol (EtOH) (1 g/kg, 20% v/v) [**(C)** *N* = 11, **(D)** *N* = 8, **(G)** *N* = 11, **(H)** *N* = 9] in the presence of reverse dialysis application in the pVTA, beginning 30 min before EtOH administration, of D-penicillamine (DP) (75 μM) **(A–D)** or 3-amino-1,2,4-triazole (3AT) (1 nM) **(E–H)** on pVTA salsolinol **(A,C,E,G)** and on ipsilateral and contralateral AcbSh DA **(B,D,F,H)** dialysates. Horizontal bars depict the contents of the pVTA perfusion fluid along the experiments. Vertical arrows indicate the last pVTA or AcbSh microdialysis sample before vehicle or EtOH administration. Filled symbols indicate samples representing *p* < 0.001 vs. basal; ^∗^*p* < 0.01 vs. vehicle administration; ^x^*p* < 0.01 vs. ipsilateral area.

As shown in Figure 4, pVTA perfusion with DP fails, also after vehicle administration, to affect the concentration of salsolinol in pVTA dialysates (Figure 4A) as well as those of DA in the ipsilateral and contralateral AcbSh (Figure 4B). However, pVTA perfusion with DP affects both ethanol-induced formation of salsolinol in the pVTA (Figure 4C) and stimulation of DA release in the ipsilateral, but not in the contralateral, AcbSh (Figure 4D).

Two-way ANOVA of salsolinol concentrations in the presence of DP perfusion of the pVTA failed to reveal significant effects of treatment (EtOH vs. vehicle) [*F*_(1_, _15)_ = 1.09; *p* = 0.3] and time [*F*_(12_, _180_ = 0.72; *p* = 0.7] and a significant treatment × time interaction [*F*_(12_, _180)_ = 0.72; *p* = 0.73]. Moreover Friedman’s ANOVA and Kendall’s coefficient of concordance analysis resulted in χ^2^_(__*N* = 17_, _*df* = 12)_ = 24.76, *p* < 0.02; coeff. of concordance = 0.12; average rank *r* = 0.66.

Three-way ANOVA of DA data showed a significant effect of treatment (EtOH vs. vehicle) [*F*_(1_, _12)_ = 11.4; *p* < 0.005] and time [*F*_(12_, _144)_ = 2.98; *p* < 0.001] and the following significant interactions: probe placement × time [*F*_(12_, _144)_ = 2.75; *p* < 0.005], treatment × time [*F*_(12_, _144)_ = 4.42; *p* < 0.001], and probe placement × treatment × time [*F*_(12_, _144)_ = 2.35; *p* < 0.008]. Tukey’s *post hoc* analysis revealed that DP significantly prevents both pVTA salsolinol formation and ipsilateral AcbSh DA increases with respect to basal values (*p* < 0.05).

Similarly, pVTA perfusion with 3AT fails, also after vehicle administration, to affect the concentration of salsolinol in pVTA dialysates (Figure 4E) as well as those of DA in the ipsilateral and contralateral AcbSh (Figure 4F). However, pVTA perfusion with 3AT affects both ethanol-induced formation of salsolinol in the pVTA (Figure 4G) and stimulation of DA release in the ipsilateral, but not contralateral, AcbSh (Figure 4H).

Two-way ANOVA of salsolinol concentrations in the presence of 3AT perfusion of the pVTA failed to reveal significant effects of treatment (EtOH vs. vehicle) [*F*_(1_, _15)_ = 1.15; *p* = 0.3] and time [*F*_(12_, _180)_ = 0.67; *p* = 0.77] and also a significant treatment × time interaction [*F*_(12_, _180)_ = 0.71; *p* = 0.78]. Moreover, Friedman’s ANOVA and Kendall’s coefficient of concordance analysis resulted in χ^2^_(__*N* = 17_, _*df*_ = _12)_ = 15.91, *p* < 0.19; coeff. of concordance = 0.078; average rank *r* = 0.02.

Three-way ANOVA of DA data revealed a significant effect of treatment (EtOH vs. vehicle) [*F*_(1_, _13)_ = 7.52; *p* < 0.02] and time [*F*_(12_, _156)_ = 3.37; *p* < 0.001] and the following significant interactions: probe placement × time [*F*_(12_, _156)_ = 2.50; *p* < 0.005], treatment × time [*F*_(12_, _156)_ = 4.88; *p* < 0.001], and probe placement × treatment × time [*F*_(12_, _156)_ = 3.14; *p* < 0.001]. Tukey’s *post hoc* analysis revealed that 3AT significantly prevents both ethanol-induced increases of pVTA salsolinol and stimulation of ipsilateral AcbSh DA release with respect to basal values (*p* < 0.05). In addition, two-way ANOVA of contralateral AcbSh DA data (shown, respectively, in Figures 3D, 4D,H) disclosed a significant effect of time [*F*_(12_, _168)_ = 17.68; *p* < 0.0001] but not of treatment [*F*_(2_, _14)_ = 0.81; *p* > 0.005] nor a significant treatment × time interaction [*F*_(24_, _168)_ = 1.23; *p* > 0.005].

**FIGURE 5:**
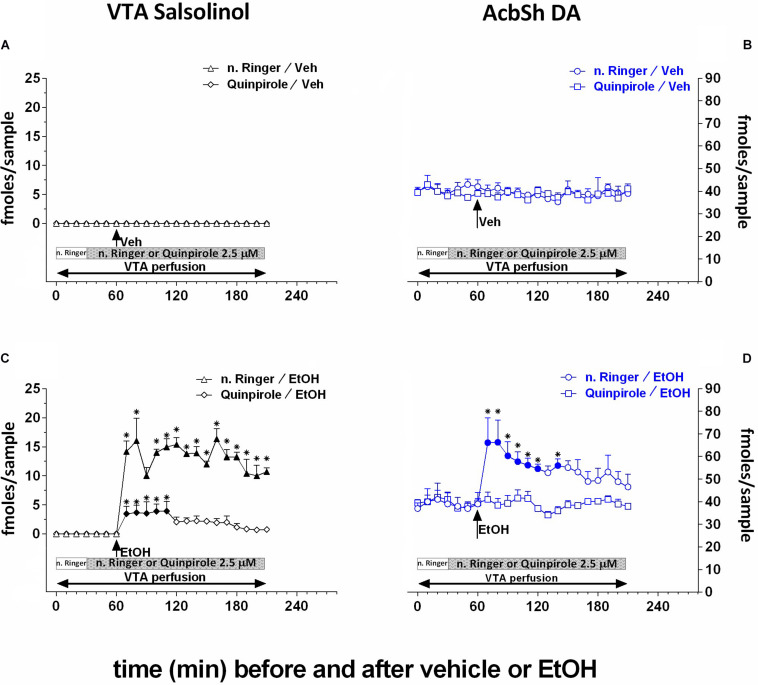
Effects of intragastric administration of vehicle (tap water, 10 ml/kg) [**(A)** *N* = 7, **(B)** *N* = 8] or ethanol (EtOH) (1 g/kg, 20% v/v) [**(C)** *N* = 9, **(D)** *N* = 8] in the presence of reverse dialysis application in the pVTA, beginning 30 min before vehicle or EtOH administration, of normal Ringer (*n*. Ringer) or quinpirole (2.5 μM) on pVTA salsolinol [**(A,C)**; triangles: *n*. Ringer; lozenges: quinpirole] and on ipsilateral AcbSh DA dialysates [**(B,D)**; circles: *n*. Ringer; squares: quinpirole]. Horizontal bars depict the contents of the pVTA perfusion fluid along the experiment. Vertical arrows indicate the last pVTA or AcbSh microdialysis sample before vehicle or EtOH administration. Filled symbols indicate samples representing *p* < 0.001 vs. basal; *p < 0.05 vs. vehicle.

Moreover, as shown in Figure 5, by perfusing the pVTA with the DA D_2_/D_3_ receptor agonist, quinpirole (2.5 μM), we also exploited the ability of D_2_/D_3_ autoreceptor stimulation to prevent DA availability for condensation with acetaldehyde to generate salsolinol. Quinpirole does not affect the concentration of salsolinol in pVTA dialysates (Figure 5A) as well as that of DA in the ipsilateral AcbSh (Figure 5B). However, after ethanol administration, pVTA perfusion with quinpirole affects salsolinol formation in the pVTA (Figure 5C) and DA release in the ipsilateral AcbSh (Figure 5D).

Two-way ANOVA of salsolinol concentrations showed significant effects of treatment (EtOH vs. vehicle) [*F*_(1_, _6)_ = 11.01; *p* < 0.02] and time [*F*_(18_, _108)_ = 3.06; *p* < 0.001] and a significant treatment × time interaction [*F*_(18_, _108)_ = 3.06; *p* < 0.001]. Moreover, Friedman’s ANOVA and Kendall’s coefficient of concordance analysis resulted in χ^2^_(__*N* = 10_, _*df* = 18)_ = 44.16, *p* < 0.00055; coeff. of concordance = 0.24; average rank *r* = 0.16.

Two-way ANOVA of DA data failed to reveal significant effects of treatment (EtOH vs. vehicle) [*F*_(1_, _6)_ = 0.07; *p* = 0.8] and time [*F*_(12_, _72)_ = 1.08; *p* = 0.4] and a significant treatment × time interaction [*F*_(12_, _72)_ = 1.69; *p* = 0.09]. Tukey’s *post hoc* analysis revealed that quinpirole significantly prevents both ethanol-induced increase of salsolinol in the pVTA and DA release in the ipsilateral AcbSh with respect to basal values (*p* < 0.05).

### Effects of pVTA μOR Blockade on pVTA Salsolinol and Ipsilateral and Contralateral AcbSh DA

**FIGURE 6:**
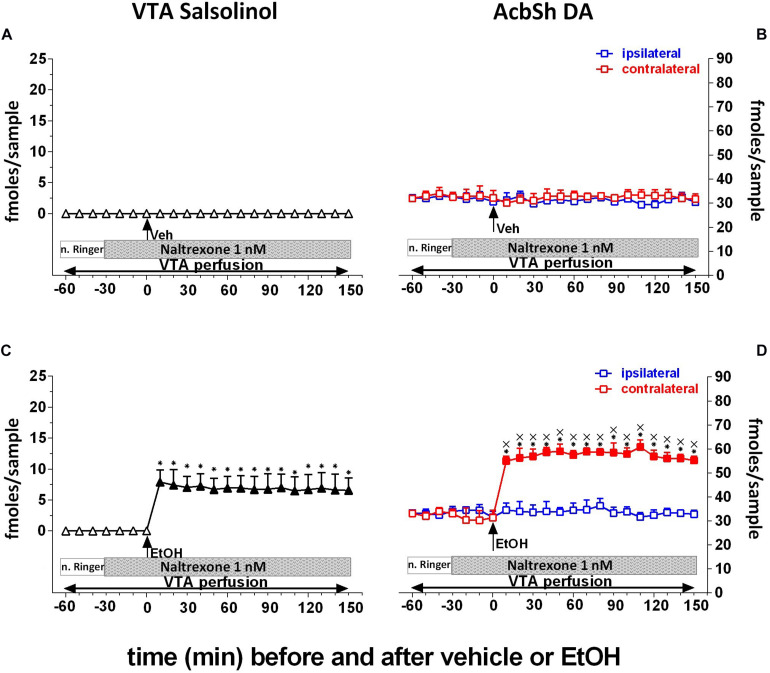
Effects of intragastric administration of vehicle (tap water, 10 ml/kg) [**(A)** *N* = 9, **(B)** *N* = 8] or ethanol (EtOH) (1 g/kg, 20% v/v) [**(C)** *N* = 12, **(D)** *N* = 11] in the presence of reverse dialysis application in the pVTA, beginning 30 min before EtOH administration, of naltrexone (1 nM) on VTA salsolinol **(A,C)** and on ipsilateral and contralateral AcbSh DA **(B,D)** dialysates. Horizontal bars depict the contents of the pVTA perfusion fluid along the experiment. Vertical arrows indicate the last pVTA or AcbSh microdialysis sample before vehicle or EtOH administration. Filled symbols indicate samples representing *p* < 0.001 vs. basal; *p < 0.01 vs. vehicle administration; ^x^*p* < 0.01 vs. ipsilateral area.

Finally, to test the hypothesis that ethanol’s effects on AcbSh DA release are mediated by the ability of ethanol-derived salsolinol to stimulate pVTA μORs, naltrexone (1 nM) was applied by reverse dialysis to the pVTA. Figure 6 shows that naltrexone, also after vehicle administration, does not affect the concentration of salsolinol in pVTA dialysates (Figure 6A) as well as those of DA in the ipsilateral and contralateral AcbSh (Figure 6B). Moreover, after ethanol administration, naltrexone does not affect the generation of salsolinol in the pVTA (Figure 6C) but affects the ability of ethanol to stimulate DA release in the ipsilateral, but not contralateral, AcbSh (Figure 6D).

Two-way ANOVA of salsolinol concentrations revealed a significant effect of treatment (EtOH vs. vehicle) [*F*_(1_, _17)_ = 595.18; *p* < 0.001] and time [*F*_(15_, _255)_ = 45.14; *p* < 0.001] and a significant treatment × time interaction [*F*_(15_, _255)_ = 45.06; *p* < 0.001]. Moreover, Friedman’s ANOVA and Kendall’s coefficient of concordance analysis resulted in χ^2^_(__*N* = 19_, _*df* = 18)_ = 55.45, *p* < 0.00001; coeff. of concordance = 0.16; average rank *r* = 0.12.

Three-way ANOVA of DA data failed to reveal significant effects of treatment (EtOH vs. vehicle) [*F*_(1_, _9)_ = 0.35; *p* = 0.57], probe placement [*F*_(1_, _9)_ = 0.58; *p* = 0.46], and time [*F*_(12_, _108)_ = 0.94; *p* = 0.51] and significant treatment × probe placement [*F*_(1_, _9)_ = 2.75; *p* = 0.13], treatment × time [*F*_(12_, _108)_ = 1.48; *p* = 0.14], probe placement × time [*F*_(12_, _108)_ = 1.34; *p* = 0.2], and treatment × probe placement × time [*F*_(12_, _108)_ = 0.96; *p* = 0.5] interactions.

Tukey’s *post hoc* analysis revealed that naltrexone significantly prevents ethanol-induced increase of ipsilateral AcbSh DA release with respect to basal values (*p* < 0.05) while leaving unaffected the formation of salsolinol in the pVTA.

## Discussion

**FIGURE 7:**
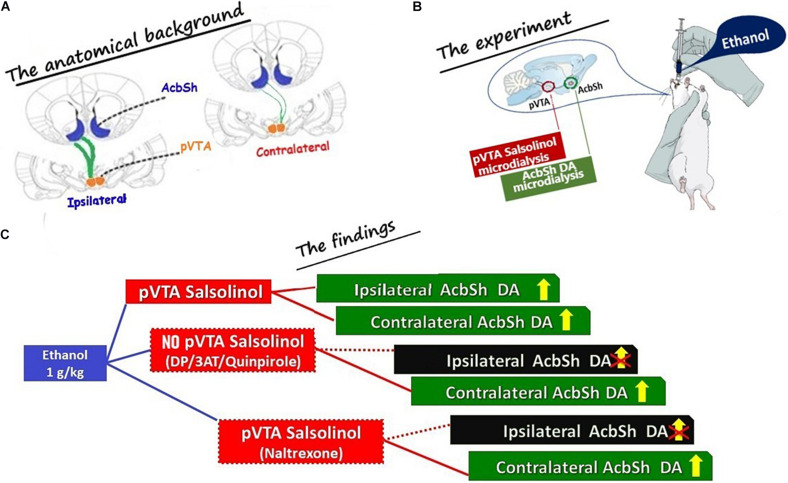
Schematic drawing summarizing **(A)** the anatomical background exploited during the microdialysis experiments (*The anatomical background*); **(B)** the main experimental conditions (*The experiment*): (i) ethanol was administered systemically, (ii) salsolinol was sampled from the posterior ventral tegmental area (pVTA), and (iii) DA was sampled from the shell of the nucleus accumbens (AcbSh) of the same (ipsilateral) or of the opposite (contralateral) side with respect to the pVTA; and **(C)** the main results (*The findings*; shown in Figures 3–6). The projections from the pVTA to the AcbSh of the same side (ipsilateral) are shown as thick green lines to indicate that these are prevailing for > 95% (Jaeger et al., 1983; Geisler and Zahm, 2005; Ikemoto, 2007; Breton et al., 2019) on those aiming at the AcbSh of the opposite side (contralateral) shown as green thin lines to indicate that these are only a minority (<5%). Dotted red lines are used in **(C)** to “connect” the box indicating the pVTA where the formation of salsolinol was prevented (DP or 3AT or quinpirole) and indicating the ipsilateral AcbSh where DA release was not stimulated by systemic ethanol. Similarly, a dotted red line is also used in **(C)** to “connect” the box indicating the pVTA, where there is formation of salsolinol and μ opioid receptors are blocked by naltrexone, and indicating the ipsilateral AcbSh where DA release was not stimulated by systemic ethanol.

The present study aimed at challenging, *in vivo*, the hypothesis that ethanol activates the mesolimbic DA system acting as the prodrug of salsolinol. To directly test this hypothesis (Polache and Granero, 2013; Peana et al., 2016), we envisioned to detect salsolinol and DA through microdialysis probes implanted in the pVTA and AcbSh, respectively, of the same side (ipsilateral) or of the opposite side (contralateral) of the rat brain, taking advantage of the mostly ipsilateral VTA–AcbSh projections (Geisler and Zahm, 2005; Ikemoto, 2007; Breton et al., 2019). In this regard, two anatomical details enforce some attention: the first one refers to the anteroposterior heterogeneity of the VTA. This has been recently in-depth reviewed (Sanchez-Catalan et al., 2014) disclosing that the ability of ethanol (but also of acetaldehyde and salsolinol) to sustain its intracranial self-administration and elicit locomotor activation resides in the posterior, but not anterior, portion of this brain structure (Rodd-Henricks et al., 2000, 2002; Rodd et al., 2004, 2005, 2008; Ding et al., 2009, 2012; Sánchez-Catalán et al., 2009; Hipólito et al., 2010, 2011; Marti-Prats et al., 2010, Martí-Prats et al., 2013). For this reason, in the present experiments, the microdialysis probes were implanted in the pVTA at brain stereotaxic coordinates in agreement with the above studies. Also, it is indeed critical, in this context, to point out that since over 95% of pVTA projections to AcbSh are ipsilateral, second anatomical detail that enforces attention (Figure 7A), our study exploited this anatomical peculiarity to reach the demonstration that reveals something that had long been suggested by indirect evidence but never proven: the until now unknown mechanism by which ethanol stimulates DA release in the AcbSh and exerts the potential to trigger its addictive liability. The present results confirmed that the oral administration of a dose of ethanol responsible of a BEC that produces mild euphoriant effects in humans (Majchrowicz, 1975; Gill et al., 1986; Nurmi et al., 1994) increases DA release in the AcbSh (Howard et al., 2008; Bassareo et al., 2019). In addition, the results disclose for the first time that this systemic administration of ethanol also determines the appearance of salsolinol, a molecule undetectable in pVTA dialysates under control conditions, no matter whether in dialysates of the same or of the opposite side with respect to that where we could simultaneously detect the increase of AcbSh DA release. However this ethanol-dependent appearance of salsolinol in pVTA dialysates, although representing on its own the first *in vivo* demonstration of the long-sought evidence (Polache and Granero, 2013) of the ethanol-dependent formation of salsolinol in the pVTA, still necessitated to be causally linked to the ethanol-dependent increase of AcbSh DA release (Polache and Granero, 2013; Peana et al., 2016). Hence, we firstly sought to determine whether, in agreement with the mostly ipsilateral VTA–AcbSh projections (Jaeger et al., 1983; Geisler and Zahm, 2005; Ikemoto, 2007; Breton et al., 2019), the application of salsolinol in the pVTA of one side would result in increased DA release only in the ipsilateral AcbSh. In agreement with previous studies (Hipólito et al., 2009, 2011), salsolinol, applied by reverse dialysis in the pVTA, significantly increases DA release in the ipsilateral AcbSh but does not affect it in the AcbSh of the opposite side. Notably, given the small percentage of pVTA neurons that project to the AcbSh of the opposite side (Jaeger et al., 1983; Geisler and Zahm, 2005), a detection system such as fast-scan cyclic voltammetry, endowed with greater analytical and temporal resolution, might have allowed to detect similar effects also contralaterally. However, the analytical sensitivity (5 fmol/10 μl) and the relatively low temporal resolution of sample collection of our assay prevented to find similar effects also in the contralateral AcbSh. In this regard, this apparent analytical limitation provided us, on the contrary, the optimal experimental conditions to test our working hypothesis. Thus, to temporally and mechanistically lock ethanol-dependent pVTA salsolinol to ethanol-dependent increases of AcbSh DA release, we sought to interfere with the formation of salsolinol. To this end, according to a common and validated approach of the studies aimed at assessing the role of acetaldehyde in ethanol’s central effects (Spivak et al., 1987; Pastor et al., 2002; Correa et al., 2012; Martí-Prats et al., 2013; Peana et al., 2017a), we prevented acetaldehyde’s bioavailability by blocking catalase-mediated ethanol metabolism with 3AT or by sequestering acetaldehyde with DP (Quertemont et al., 2003; Font et al., 2006a, b; Correa et al., 2008; Peana et al., 2015). The results of these experiments demonstrate that without acetaldehyde formation and/or availability in the pVTA, there is no detectable salsolinol in pVTA dialysates nor increased DA release in the ipsilateral AcbSh after ethanol’s systemic administration. In contrast, in the AcbSh of the side opposite to that where pVTA was reverse applied with 3AT or DP, systemic ethanol significantly increases DA release. Thus, based on previous observations in the ipsilateral pVTA and AcbSh, we can conclude that the increases of DA release in the contralateral AcbSh are further indirect proof of the newly formed salsolinol-dependent increase of AcbSh DA release. In this regard, it is relevant to observe that salsolinol was detected at a concentration in the low nM range (∼15 fmol/10 μl, i.e., 1.5 nM), very similar to that (10 nM) used in the experiments in which salsolinol, reverse applied in the pVTA (Figure 3), increases DA release in the ipsilateral AcbSh. Notably, in agreement with the observation that no catalase staining could be found in the nucleus accumbens (Zimatkin and Lindros, 1996), we could not detect salsolinol in AcbSh dialysates (Figure 2).

In summary, the present *in vivo* results (Figure 7) point to the catalase-mediated metabolic conversion of ethanol into acetaldehyde in the pVTA and to the subsequent condensation of acetaldehyde with DA to generate salsolinol, as key mechanisms for the ability of ethanol to excite DA neurons and, consequently, to stimulate DA release in the AcbSh (Di Chiara et al., 2004; Howard et al., 2008; Bassareo et al., 2017, 2019; Volkow et al., 2017; Wise and Robble, 2020).

Previous extensive literature indicates that the reduced activity of DA cells in the posterior, but not anterior, VTA (Rodd et al., 2004, 2008) by local injections of quinpirole can reduce ethanol intake (Nowak et al., 2000) and both ethanol (Hodge et al., 1993; Hauser et al., 2011) and salsolinol (Rodd et al., 2008) self-injections in the pVTA. Hence, since salsolinol is formed by DA and acetaldehyde (Davis and Walsh, 1970; Davis et al., 1970) no matter, in this context, whether spontaneously (by Pictet–Spengler condensation) or enzymatically (Chen et al., 2018), we also addressed the possibility of exploiting the reduction of synaptic availability of DA in the pVTA, by reverse application of quinpirole, to further challenge our working hypothesis. The results of this experiment (Figure 5) disclose that, after ethanol’s systemic administration, the stimulation of pVTA DA D_2_/D_3_ autoreceptors at a concentration in the range that prevents ethanol (Rodd et al., 2004; Hauser et al., 2011) and salsolinol (Rodd et al., 2008) self-administration does not affect basal AcbSh DA release but significantly reduces the formation of salsolinol in the pVTA and prevents the ethanol-elicited increase of DA release in the ipsilateral AcbSh. These results rule out the possibility that the effects of ethanol in the pVTA could be directly mediated by acetaldehyde instead of salsolinol and are in agreement with our recent report that in the absence of newly synthesized DA, unlike salsolinol, neither ethanol nor acetaldehyde can stimulate pVTA DA neurons recorded in mesencephalic, pVTA containing, slices (Melis et al., 2015). Moreover, while the effect of ethanol on the firing of pVTA DA neurons in slices from α-methyl-p-tyrosine-treated mice could be restored by the application of exogenous DA, this was blocked by catalase inhibition (Melis et al., 2015). Notably, although after ethanol administration in the presence of pVTA perfusion with quinpirole we could still detect a significant formation of salsolinol (Figure 5C), this was significantly lower in comparison with that determined in the presence of pVTA perfusion with normal Ringer. Moreover, although detectable (Figure 5C), such salsolinol’s concentration was insufficient to trigger AcbSh DA release (Figure 5D). A possible interpretation of this finding might also be that pVTA perfusion with quinpirole, at the concentration that on its own does not affect basal AcbSh DA release, may have exerted a further preventive action (Rodd et al., 2004, 2008; Hauser et al., 2011) on the stimulatory effect of ethanol-derived salsolinol in the pVTA (Hipólito et al., 2009, 2011; Melis et al., 2015).

The present data also point to μORs as those through which the ethanol-derived, newly formed, salsolinol stimulates DA neurons in the pVTA (Figure 6). This is consistent with *ex vivo* and *in vivo* evidence that μOR blockade prevents salsolinol-induced excitation of DA neurons (Xie et al., 2012), DA release in the Acb (Hipólito et al., 2009, 2011), locomotor activation (Hipólito et al., 2010), and conditioned place preference (Matsuzawa et al., 2000; Hipólito et al., 2011; Campos-Jurado et al., 2020). In this regard, the present study provides a mechanistic framework for naltrexone as FDA-approved treatment for alcohol use disorder (AUD) (American Psychiatric Association, 2013).

However, apparently at variance with both the present and previous data (Melis et al., 2015), it has recently been proposed that KCNK13 potassium channels may be the molecular target for the stimulatory action of ethanol on pVTA DA neurons and for ethanol-dependent behaviors (You et al., 2019). Nevertheless, this does not preclude the possibility that these effects are mediated through stimulation of μORs by ethanol-derived salsolinol. In this regard, it would be critical to test, in brain slices from α-methyl-p-tyrosine-treated animals, the suggestion put forward by You et al. (2019).

In conclusion, the present study demonstrates that the intragastric administration of ethanol results, in the pVTA, in pharmacologically effective concentrations of salsolinol that, *via* local stimulation of μORs, increases DA release in the AcbSh, the neurochemical mechanism potentially responsible for triggering ethanol’s abnormal consumption (Di Chiara et al., 2004; Volkow et al., 2017; Wise and Robble, 2020). However, we acknowledge that these findings would be of greater impact if also investigated on female rats. Moreover, while we also acknowledge that this is still far from conclusively linking newly formed salsolinol in the pVTA to AUD, we envision that the significance and impact of the present discovery would be greatly amplified if confirmed also under voluntary consumption by ethanol-preferring rats. Ethanol, in fact, being a molecule endowed with addictive potential, may become responsible for AUD and for its negative consequences on health (Rehm et al., 2017; Axley et al., 2019) when encounters genetic vulnerability to its excessive consumption. Hence, the disclosure of ethanol’s precise site and mechanism of action on both genders, together with the discovery of the genetic bases of proclivity to excessive ethanol consumption, will represent significant progresses not only for the scientific community but also for the organizations that, at different levels, are in charge of individual and societal healthcare policies.

In conclusion, all these limitations notwithstanding, the present demonstration validates and sustains the development of future studies devoted to further investigate the disclosed mechanism after voluntary consumption and to define preventive and therapeutic strategies based on acetaldehyde sequestration (Orrico et al., 2017) or on the use of appropriate antioxidant agents (Aragon et al., 1991).

## Data Availability Statement

The raw data supporting the conclusions of this article will be made available by the authors, without undue reservation.

## Ethics Statement

The animal study was reviewed and approved by CESA University of cagliari.

## Author Contributions

VB: methodology, investigation, writing of the original draft, and review and editing. RF and CM: methodology and investigation. RMa, SP, and PC: methodology, investigation, and writing of the original draft. AP: resources and writing of the original draft. RMi: writing of the original draft. EA: conceptualization, resources, writing of the original draft, review and editing, and supervision. All authors contributed to the article and approved the submitted version.

## Conflict of Interest

The authors declare that the research was conducted in the absence of any commercial or financial relationships that could be construed as a potential conflict of interest.
